# Postbiotic Dietary Supplementation with Sonicated *Shewanella* sp. SpPdp11 Improves Intestinal Status in Juvenile Senegalese Sole (*Solea senegalensis*)

**DOI:** 10.1007/s10126-026-10608-3

**Published:** 2026-04-14

**Authors:** I.M Cerezo, B. Delgado-Martín, A.J Vizcaíno, A. Galafat, F.J Alarcón-López, I. García de la Banda, A. Hernández de Rojas, L. Díaz, M.C Balebona, S.T Tapia-Paniagua, M. A. Moriñigo

**Affiliations:** 1 Departamento de Microbiología, Campus de Excelencia Internacional del Mar (Ceimar), Facultad de Ciencias, Universidad de Málaga, Campus de Teatinos s/n, Málaga, 29071 Spain; 2 Departamento de Biología y Geología, Campus de Excelencia Internacional del Mar (Ceimar), Universidad de Almeria, La Cañada de San Urbano, Almería, 04120 Spain; 3 Unidad de Bioinformática, Supercomputing and Bioinnovation Center (SCBI), Universidad de Málaga, C. Severo Ochoa, 34, Málaga, 29590 Spain; 4 Instituto Español de Oceanografia, Centro Oceanográfico de Santander, Santander, 39080 Spain; 5 LifeBioencapsulation S.L, Parque Científico PITA, El Alquián, Almería, 04131 Spain

**Keywords:** Postbiotic, Intestinal health, Inflammation, Gut microbiota, *Solea senegalensis*, Aquaculture, Preventive strategy

## Abstract

The use of non-viable microbial preparations (postbiotics) is emerging as a safe alternative to probiotics in aquaculture. In this study, we investigated the impact of dietary inclusion of sonicated cells of the marine bacterium *Shewanella* sp. Pdp11 (SpPdp11) on the intestinal health of juvenile Senegalese sole (*Solea senegalensis*). Fish were fed a control diet or a diet supplemented with SpPdp11 postbiotic for 45 days, and their intestinal status was assessed using histological, transcriptomic, and microbiota analyses. Postbiotic supplementation improved intestinal morphology by widening villi and reducing lamina propria and epithelial height, suggesting enhanced barrier function. RNA-seq analysis revealed downregulation of inflammatory pathways, including ECM receptor interaction and PPAR signaling, together with activation of bile acid biosynthesis. 16 S rRNA profiling showed a marked shift in microbial composition, with decreased prevalence of opportunistic taxa such as *Vibrio* and *Stenotrophomona*s and enrichment of beneficial genera, including *Acinetobacter*,* Pseudomonas*,* and Malacoplasma.* These results provide evidence that marine-derived postbiotics can modulate host physiology and gut microbial communities without the risks associated with live probiotics. Sonicated SpPdp11 represents a promising biotechnological feed additive to support intestinal health, reduce inflammation, and promote sustainable aquaculture practices.

**Supplementary Information:**

The online version contains supplementary material available at 10.1007/s10126-026-10608-3.

## Introduction

The stability of the gut is essential for optimizing growth and ensuring the health of farmed fish in aquaculture production (Zhang et al. 2020). A functional gastrointestinal system enhances nutrient absorption, digestion efficiency, and acts as a key barrier against pathogen invasion (Zhang et al. 2020 a, Benjamin et al. 2013). Disruptions to this barrier, often caused by dietary imbalances, can increase mucosal permeability, promote inflammation, and facilitate bacterial translocation, ultimately compromising fish health (Landgraf et al. 2017; Mosberian-Tanha et al. 2016).

Among the factors influencing gut health, diet plays a pivotal role in shaping the composition and function of the intestinal microbiota (Martin and Król 2017; Yukgehnaish et al. 2020). A stable gut microbiota contributes to host immunity, nutrient metabolism, and serves as a frontline defense against pathogens (Belkaid and Hand 2014; Levy et al. 2017). Intestinal microbiota is an essential biological barrier against pathogens (Brown et al. 2019) by interacting with the host’s immunological system (Tremaroli and Bäckhed 2012; Flint et al. 2014). In addition, the intestinal microbiota contribute to host health by producing beneficial metabolites that support gut function (Tremaroli and Bäckhed 2012; Levy et al. 2017). Dysbiosis, or imbalance of this microbial community, has been linked to increased susceptibility to diseases and immune disorders in fish (Rombout et al. 2010; Standen et al. 2015; Foucault et al. 2022; Mougin and Joyce 2023). For this reason, dietary interventions, particularly the use of probiotics, have emerged as preventive strategies to modulate gut microbiota and enhance fish health (Abul et al. 2018; Li et al. 2022).

Probiotics are defined as live microorganisms that, when administered in adequate amounts, confer health benefits to the host (Merrifield et al. 2010; Nayak 2020) Their viability has traditionally been considered essential for their beneficial effects (Binda et al. 2020). However, concerns about the safety, stability, and standardization of live probiotic formulations have spurred growing interest in alternative approaches (Choudhury and Kamilya 2018). In this context, postbiotics are defined as preparations of inanimate microorganisms and/or their components that confer health benefits to the host, have recently gained recognition (Salminen et al. 2021).

Postbiotics may retain many of the advantages of live probiotics, including immunomodulation and gut microbiota regulation, while offering additional benefits such as greater safety, longer shelf-life, and ease of incorporation into feeds (Wang et al. 2023a; Barui et al. 2024; Ballantyne et al. 2023; Li and Tran 2022; De Almada et al. 2016). Several inactivation methods exist to produce postbiotic formulations, including heat treatment, high pressure, UV radiation, and sonication (Alzamora et al. 2011). Among them, sonication is particularly promising for aquaculture applications, as it preserves key bioactive components (Gibson et al. 2009). Compared to thermal inactivation, sonication operates at lower temperature, which helps to preserving key bioactive components such as cell wall fragments, membrane components, proteins, polysaccharides, and other intracellular bioactive molecules that are critical for the beneficial effects of postbiotics, and then, is energy efficient and environmentally friendly, reducing both water and energy consumption during processing (De Almada et al. 2016; Starek et al. 2021). Some authors have reported that sonication-inactivated probiotic cells *Bacillus subtilis* AB1 are effective in controlling infections caused by *Aeromona*s sp. in rainbow trout (Newaj-Fyzul et al. 2007); sonicated *Lactobacillus plantarum* cells improved the anti-stress resistance in *Litopenaeus vannamei* (Zheng et al. 2017), and sonicated *Enterococcus avium* strain increased growth and survival in tilapia challenged with *Streptococcus agalactiae* (Chu et al. 2020).

Our research group has been actively involved in investigating the probiotic potential of *Shewanella sp.* Pdp11 (SpPdp11), a Gram-negative bacterium isolated from the healthy skin of *Sparus aurata* (Chabrillón et al. 2005). In its viable form, SpPdp11 has demonstrated numerous benefits in aquaculture species, including growth promotion, improved feed efficiency, immune system stimulation, and enhanced intestinal functionality (Ector Cordero et al. 2015; Tapia-Paniagua et al. 2015; Vidal et al. 2016). Given the challenges associated with live probiotics, our focus has extended to explore its potential as a postbiotic (Domínguez-Maqueda et al. 2021, 2024).

Therefore, the aim of this study was to investigate the effects of dietary supplementation with sonicated cells of SpPdp11 on the intestinal status of juvenile *S. senegalensis*. Through histological analysis, transcriptomic profiling, and microbiota sequencing, we aimed to assess whether this postbiotic approach could represent a preventive dietary strategy to improve intestinal integrity, reduce inflammation, and beneficially modulate gut microbial communities in aquaculture.

## Materials and Methods

### Cultured Probiotic Microorganism and Sonication

The probiotic strain SpPdp11 was cultured in Trypticase Soy Broth (TSB, Sigma) supplemented with a 1.5% NaCl (TSBs). The culture was incubated at 23 °C with orbital shaking until it entered the exponential growth phase (OD = 1, at 600 nm). Upon reaching this phase, cells were stored up after centrifugation at 3000 × g, 10 min, 4° C and an adjusting of OD = 1.7 (2 × 10^9^ colony-forming units (CFU)/mL) in phosphate-buffered saline (PBS, Sigma) (Tapia-Paniagua et al. 2014).

Subsequently, the bacterial suspension underwent sonication using the UP200S ultrasonic processor (Hielscher). Sonication parameters included 4 pulses of 30 s each, with an amplitude of 35 μm and a cycle setting of 1. Following, the SpPdp11 extract was centrifugated at 3000 × g for 5 min at 4 °C, with a total protein content of 0.4 µg/µL quantified by Qubit fluorometer (ThermoFisher). A streak plate was performed to check for live cells and the final SpPdp11 extract was stored at −20 °C until further use.

### Feed Preparation, Experimental Design and Sample Collection

Two diets were used in this essay. The commercial extruded feed Europa L3 (crude protein: 57%; crude fat: 16%; ash: 10%; cellulose. 0.10%; P total: 1.6%; Skretting, Burgos, Spain) was used as control diet (CTRL). Experimental diet was prepared with the sonicated bacterial cell preparations sprayed into the control feed to achieve a dose equivalent to 10^9^ CFU per gram of feed (PDP11). Experimental diets were prepared on a two-week basis and stored at 4 °C before daily use.

Eighty fish (initial weight 105 ± 5 g) were randomly distributed in 200 L tank with continuous seawater flow (17.1 ± 0.7 °C). Each feeding regimen was implemented in separate tanks per duplicate (*n* = 20 fish per tank, 40 per treatment). A two-week acclimatization period preceded the experimental phase. Then, juvenile specimens were fed control or experimental diet for 45 days, with feed offered twice a day. Fish were weighed every two weeks to adjust the daily feeding amounts to 2% of the total biomass. In addition, fish survival was systematically monitored throughout the experimental period.

Following the 45-day feeding trial, fish underwent a 24-h fasting period to eliminate any remaining feed particles prior to sampling. Subsequently, six fish per treatment were sacrificed with an overdose of clove oil (200 ppm). Whole intestines were then meticulously dissected under sterile conditions, and segments (0.5 cm) from both the anterior (proximal to the stomach) and posterior (pre-rectal) regions of the intestine were divided and preserved at − 80 °C for subsequent gene expression and intestinal microbiota analysis. In addition, 1 cm-length portions of the anterior and posterior intestine were collected for further examination under light microscopy.

### Histological Observations

For examination under light microscopy, intestinal tissue samples were fixed in phosphate-buffered formalin (4% v/v, pH 7.2) for 24 h, followed by dehydrated, and immersed in paraffin according to usual histological techniques. Samples were divided in sections, stained and observed under an optical microscope according to the protocol described by García-Márquez et al. (2022). The following parameters in H&E stained sections were measured on two cross-section per fish and intestinal segment: *villus* height (VH): *villi* were measured from their base to their distal tips. Only full finger-shaped and well-oriented *villi* were used; *villus* width (VW): distance from one side to other in the midpoint of the *villus*; epithelium height (EH): epithelial cells of different *villi* were measured from the basement membrane to the tip of their *microvilli*; *lamina propria* thickness (LP): three measurements of the *lamina propria* width were made along the *villi* (one at the base, one in the middle and one at apex of the *villi*); submucosa thickness (SM): measured from the outer to the inside limits of the submucosa layer, muscular thickness (MT): measured from the outer layer of the section until the outer layer of the submucosa; and serosa thickness (ST): measured from the out muscularis layer (30 independent measurements in 6 specimens per treatment and intestinal segment).

Results of histological analysis are reported as means ± standard deviation (*n* = 6 per treatment). The normal distribution of all data was examined using the Shapiro–Wilk test, and the homogeneity of the variances was checked with the Levene test. In instances where necessary, an arcsine transformation was applied. Treatment differences were tested using Student’s t-test. The significance level of *p* < 0.05 was considered significant. SPSS Statistics software was used for statistical analysis (SPSS Inc, IBM Company, NY, USA).

### RNA Extraction, Quality Assessment, and Sequencing Analysis

The extraction of total RNA (6 samples per condition and treatment) from anterior and posterior Senegalese sole gut samples was carried out with the TRIsure™ methodology (Qiagen, Germany), and the eluted RNA was stored at −80 °C for subsequent analysis. Quantification, integrity assessment, and library sequencing of the total RNA followed the protocol by Cerezo et al. (2023).

Raw reads were processed to eliminate sequencing adapters, poly-N regions, and low-quality reads using *fastqp* software (Chen et al. 2018). Following this, the Q20, Q30, and GC content of the clean data were computed, and all subsequent analyses were based on this clean, high-quality data.

The paired-end clean reads were mapped to the *S. senegalensis* reference transcriptome (Genome assembly IFAPA_SoseM_1, obtained from https://www.ncbi.nlm.nih.gov/datasets/genome/GCF_019176455.1; accessed on 30 september 2021) using *bowtie2* software (Langmead and Salzberg 2012). Transcripts counts were generated using *sam2counts* software (https://github.com/maureensmith/sam2counts.git*).* Differential expression analysis was implemented using the *DESeq2* Package in R. The differentially expressed transcripts (DEGs) were defined as those with an absolute log2 fold-change (logFC) value ≥ 1.3 and a alse discovery rate (FDR) < 0.05 across all study conditions. The enrichment analysis Significance for KEGG pathway, was determined with a p Benjamini-Hochberg adjusted value ≤ 0.05 using *clusterprofiler* R library. Enriched pathways were visualized using *ridgeplot* function of *ggridges* R package.

### DNA Extraction, Quality Assessment, and 16 s rDNA Sequencing Analysis

Gut samples (6 samples per condition and section) stored at − 80 °C were gradually defrosted on ice. The mucus contents were extracted by gently pressing towards the ends using a sterile object. After homogenization, 50 mg of each mucus sample was utilized for DNA extraction. DNA extraction followed a protocol based on saline precipitation with minor modifications (Martínez et al. 1998). The concentration of DNA was measured fluorometrically using the Qubit™ dsDNA HS Assay Kit (Thermo Fisher Scientific, Waltham, MA, USA), while spectrophotometric and electrophoretic methods were employed to assess the purity, quality, and integrity of the DNA.

Subsequently, 16 S rRNA gene was sequenced on the Illumina MiSeq platform (Illumina, San Diego, CA, USA) using 2 × 250 bp paired-end sequencing at the Ultrasequencing Service of Novogene Europe (Cambridge, United Kingdom). The protocol for the preparation of 16 S metagenomic sequencing library was followed, focusing on the V3-V4 variable regions of the 16 S rRNA gene. Sequencing was performed using the direct primer (5′ CCTAYGGGRBGCASCAG 3′) and the reverse primer 806R (5′ GGACTACNNGGGTATCTAAT 3′) (Klindworth et al. 2013).

All Illumina reads underwent analysis with FastQC software (version 0.11.4) and Q30 score was maintained at ≥ 92%. Further data processing involved trimming, 16 S analysis, and visualization using a workflow based on the DADA2 package of R software following the procedure described by Cerezo et al. (2023), using SILVA taxonomy release 138.2 (2013). Alpha diversity was estimated using the Observed, Shannon and Simpson index, and beta diversity was shown with NMDS representation. Statistical analyses between diversity indexes were performed using t-test (*p* < 0.05) and ANOSIM test was used to evaluate the changes produced by adding a postbiotic in betadiversity index (*p* < 0.05). Differential abundance of taxa was determined using DESeq2 stadistical (*p* < 0.05).

## Results

No fish mortality was recorded during the experimental period. Although growth performance was not a primary objective, the mean final body weight was 115.7 ± 5.9 g in the control group and 118.2 ± 6.3 g in the group treated with the postbiotic PDP11, with no significant differences between the groups (*p* > 0.05).

### Histological Examination of in Vivo Response

**Fig. 1:**
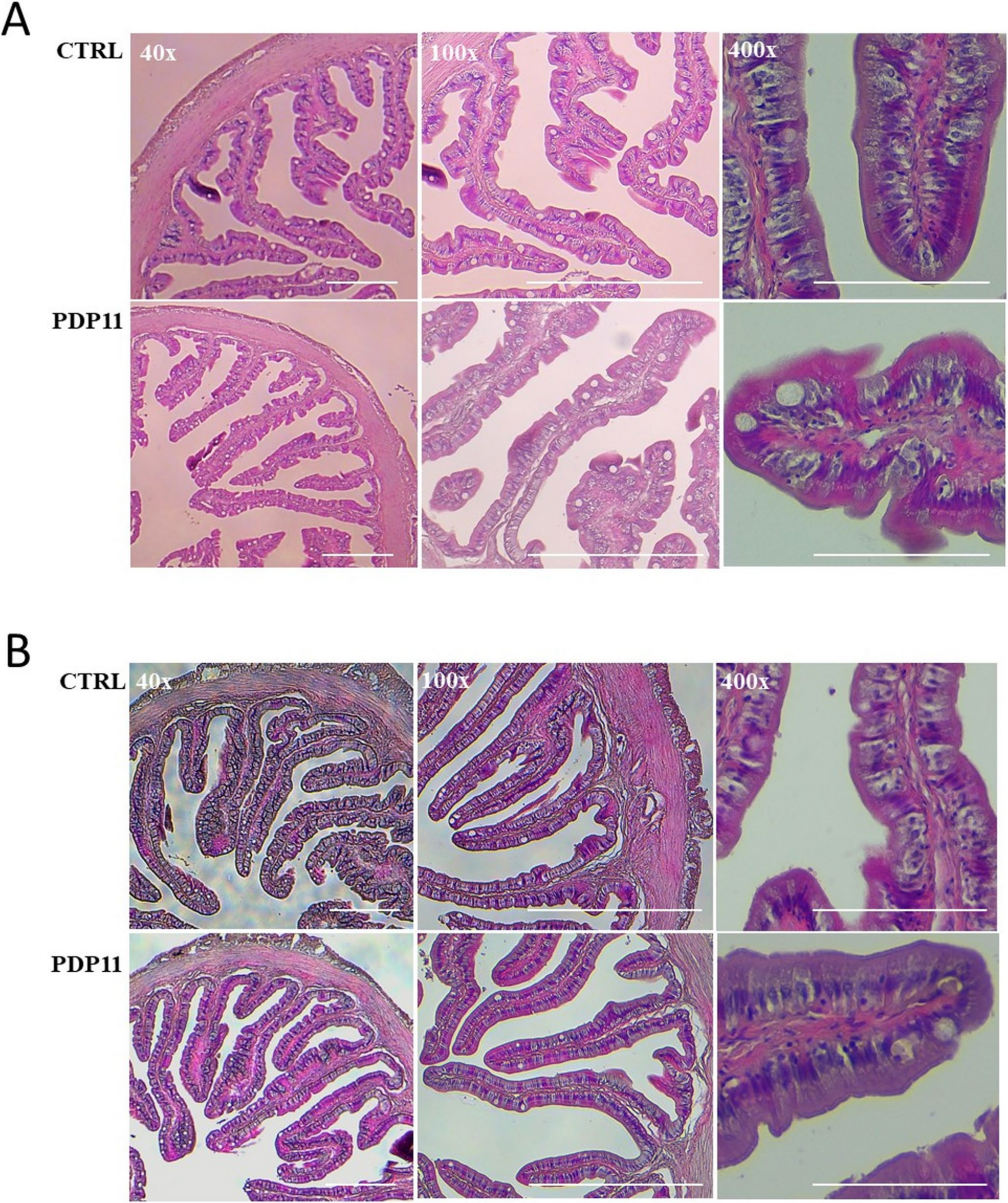
Microscopic view of (**A**) anterior and (**B**) posterior intestine from *S. senegalensis* specimens fed with CTRL and PDP11 diets, stained with Hematoxylin and Eosin. Images, taken at different magnification, per treatment (from left to right; 40x, 100x and 400x). Scale bars are provided at the bottom of each image for reference and represent 200, 200 and 50 μm in 40x, 100x and 400x, respectively

Histological sections were prepared for the anterior and posterior intestine sections to assess the effects on two diets assayed on these tissues and to infer the overall health status of the specimens. In all samples, it was evident that both segments of the intestine exhibit a normal appearance without histological alterations (Fig. 1A and B). The normal structure and organization of the intestinal *villi*, often referred to as ‘fingers in a glove’, remain intact. The nuclei of the enterocytes are consistently aligned in the basal region of the cells, while the apical part reveals an intact and continuous ‘brush border’, the superficial layer of the intestinal mucosa. These observations collectively confirm that the intestinal mucosa maintains a normal and apparently healthy structure across all animals (Fig. 1A and B).

Additionally, morphometric measurements were performed on histological images to provide a more comprehensive understanding of the intestinal structure. Table 1 summarizes the results obtained for both the anterior and posterior regions of the intestine. Concerning the *villus* height (VH), fish fed PDP11 diet did not show significant differences regard to control fish. However, there was a significant increase in *villus* width (VW) of anterior intestine in fish fed the PDP11 diet compared to the CTRL group. Serosa (SE), *muscularis* (MU) and submucosa (SM) thickness did not show significant differences among dietary treatments. On the contrary, the *lamina propria* thickness (LP), exhibited a statistically significant reduction in fish fed with postbiotic-supplemented diet. Additionally, the epithelium height (EH) was also significantly lower in the PDP11 group (Table 1).

**Table 1:** Results of morphometric analysis conducted on anterior and posterior intestine sections from *S. senegalensis* specimens fed with CTRL and PDP11 diets. Data are presented as the mean with standard deviation (*n* = 6). Significant differences (*p* < 0.05) are denoted by *

Parameter (µm)	Anterior intestine		Posterior intestine	
	CTRL	PDP11	CTRL	PDP11
VH	320.35 ± 51.56	347.44 ± 83.80	276.60 ± 83.90	282.59 ± 74.92
VW	42.72 ± 7.27	54.41 ± 10.01*	44.02 ± 12.14	47.15 ± 11.23
SE	33.50 ± 7.55	26.48 ± 7.41	32.21 ± 10.97	32.17 ± 15.98
MU	47.44 ± 12.80	54.62 ± 17.61	38.27 ± 9.01	42.96 ± 16.72
SM	15.36 ± 5.20	18.082 ± 5.96	19.66 ± 7.90	15.40 ± 3.04
LP	10.13 ± 0.39	8.08 ± 1.39*	11.84 ± 0.84	8.88 ± 0.43*
EH	15.10 ± 3.12	12.52 ± 1.87*	12.74 ± 2.34	11.10 ± 2.19*

Dietary codes: CTRL: control diet; PDP11: diet supplemented with sonicated cells of the SpPdp11 strain. *Villus* height (VH); *villus* width (VW); serosa thickness (SE); *muscularis* thickness (MU); submucosa thickness (SM); *lamina propria* thickness (LP); epithelium height (EH)

### Intestine *S. senegalensis* Transcriptome Response

RNA-seq analysis to gain a comprehensive understanding of the transcriptomic alterations in the intestine of *S. senegalensis* resulting from the dietary supplementation of sonicated probiotic SpPdp11 was carried out. Illumina sequencing yielded a total of 929,576,663 raw reads. The final read count per individual ranged from 42,253,485 to 3,309,528 with an average mapping rate of 53.00% ± 3.01% (means ± standard deviation).

**Fig. 2:**
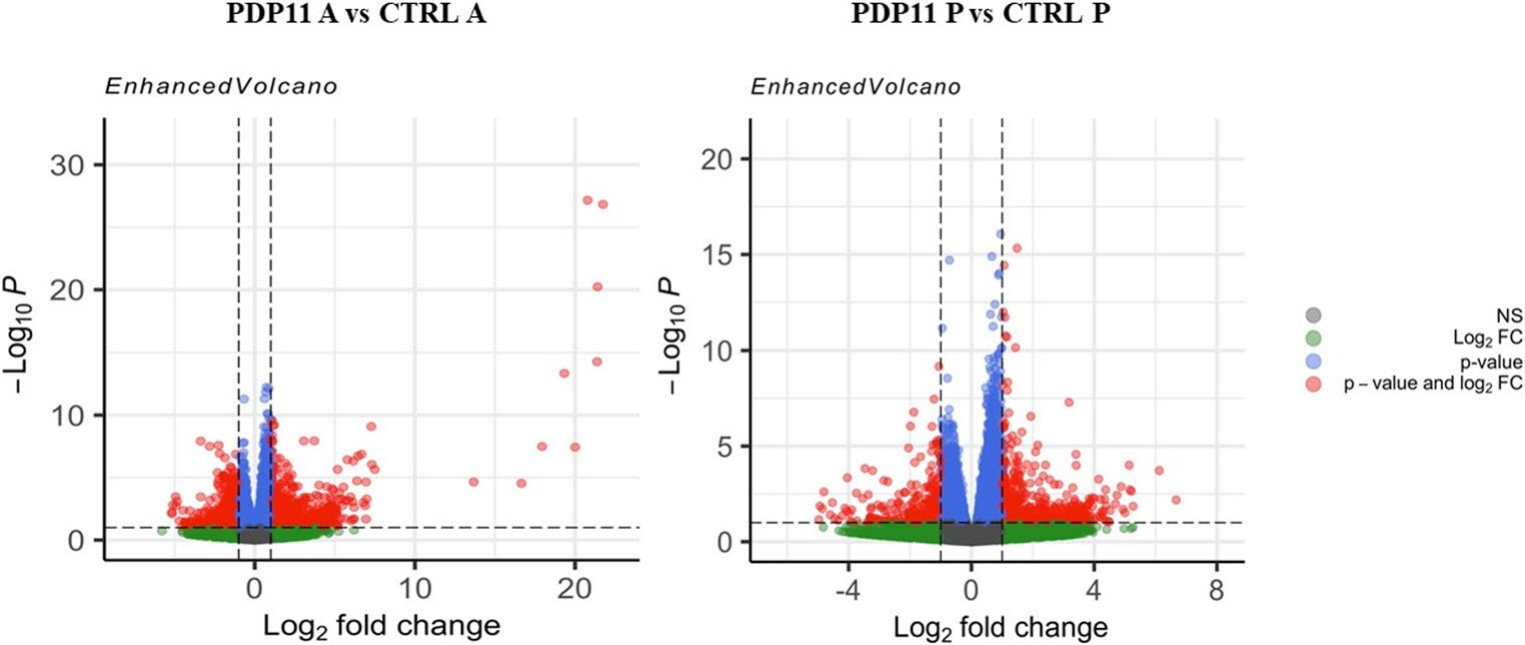
Volcano plot illustrates gene expression differences between conditions. X-axis represents Log_2_ fold change (Log_2_ FC), the Y-axis depicts statistical significance (-Log_10_ *P*-value). Grey dots represent genes that are not statistically significant (NS). Green dots represent genes that meet the Log_2_ FC threshold but not the p-value criterion for significance. Blue dots represent genes that pass the p-value criterion but not the Log_2_ FC threshold. Dots in red that are in the upper right or left corners, represent signify genes with significant expression changes (DEGs)

To identify Differentially Expressed Genes (DEGs), the RNA-seq profiles obtained from anterior and posterior intestinal sections *S. senegalensis* specimens between assayed group were compared. Only the results confirmed by *DESeq*2 with a |Log2FC| > 1.3 and p value < 0.05, were considered, resulting in a total of 247 DEGs in the anterior section and 197 DEGs in the posterior section (Fig. 2).

**Fig. 3:**
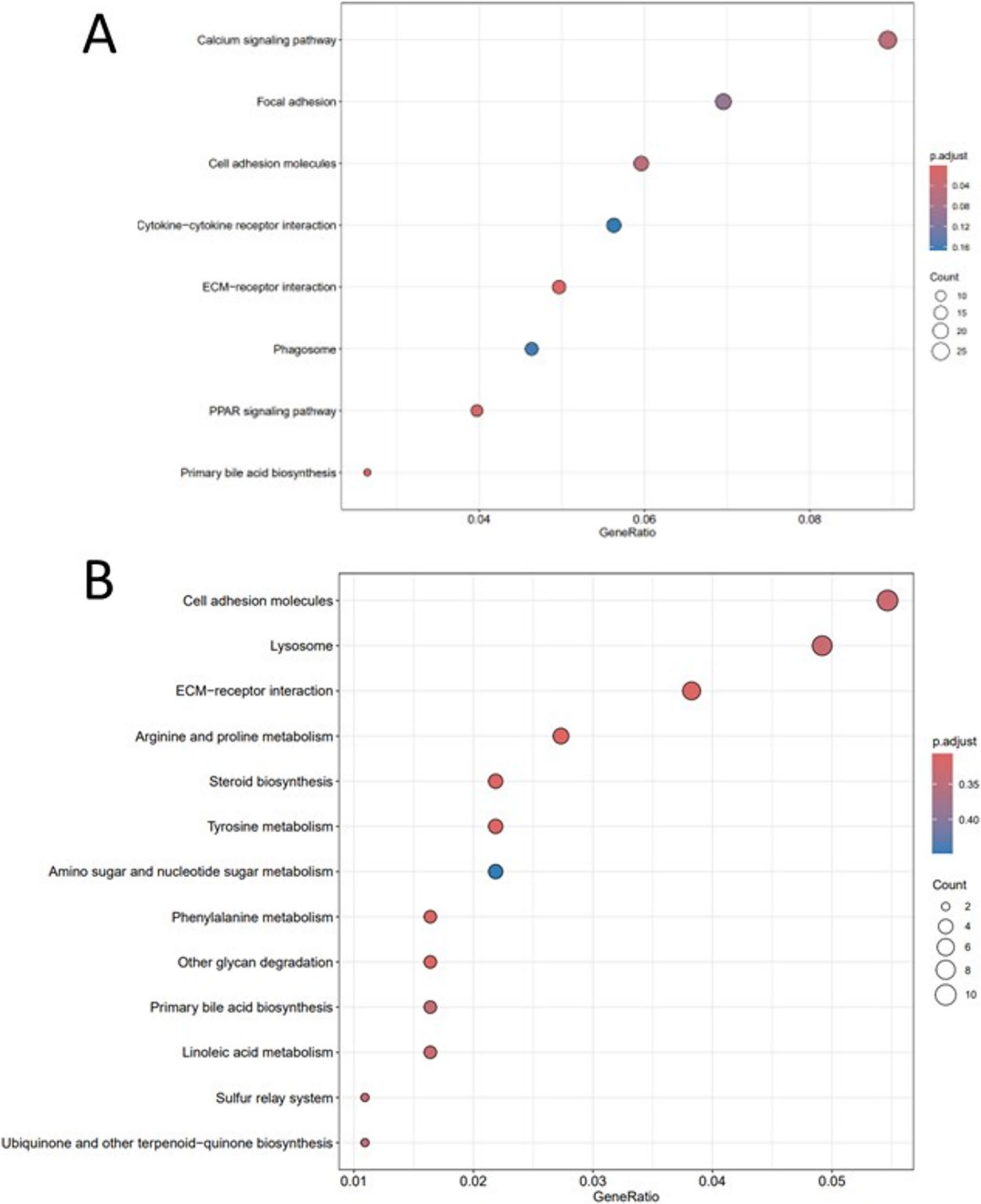
Dot plot illustrating functional enrichment results comparing *S. senegalensis* fed control (CTRL) and sonicated SpPdp11 supplementation (PDP11) in the (**A**) anterior and (**B**) posterior intestinal tracts. The horizontal axis represents the gene ratio, the vertical axis displays the KEGG pathways, and colours indicate p-values

Following the identification of DEGs, a functional enrichment analysis was conducted to assess potential enriched functions within these comparative sets. In the anterior section, when comparing sonicated SpPdp11 -fed fish to the control group, significant differences in expression of genes associated with primary bile acid biosynthesis, peroxisome proliferator-activated receptor (PPAR) signaling pathways and extracellular matrix (ECM) receptor interaction were observed (Fig. 3A). While the treatment group prominently displays overexpression of genes associated with the primary bile acid biosynthesis pathway (Supplementary Fig. 1) and suppression of the ECM receptor interaction (Supplementary Fig. 2), the trend within the PPAR signaling pathways is less evident (Supplementary Fig. 3).

However, although the routes were enriched in the posterior intestine tissue analysis, differences were not statistically significant (*p* < 0.05) (Fig. 3B).

### Intestinal Microbiota Composition

The sequences generated an average of 83,072.5 reads, ranging from 52,134 to 176,457 reads for both the anterior and posterior intestinal samples. These reads were subsequently clustered into a total of 8,654 ASVs.

The alpha diversity metric was employed to quantify diversity (Table 2). The results from the alpha diversity analysis revealed statistically significant differences (*p* < 0.05) in Observed and Shannon index in the comparison between CTRL and PDP11 groups both in anterior and posterior intestinal sections. In both cases, the control specimens showed significantly higher values than those fed the postbiotic-supplemented diet. However, Simpson index did not show significant differences regarding section or treatment.

**Table 2:** Alpha diversity of anterior (A) and posterior (P) intestinal microbiota of Senegalese sole specimens fed Control (CTRL) and sonicated probiotic SpPdp11 supplemented (PDP11) diets. * Represents significant differences (t test, *p* < 0.05) between feeding treatments in the same section

CTRL A	Observed	Shannon	Simpson
	666,60 ± 55,43*	4,20 ± 0,18*	0,94 ± 0,01
PDP11 A	604,00 ± 140,04	3,24 ± 0,56	0,88 ± 0,09
CTRL P	556,00 ± 231,40*	3,41 ± 1,01*	0,84 ± 0,18
PDP11 P	410,83 ± 66,64	2,63 ± 0, 72	0,78 ± 0,16

**Fig. 4:**
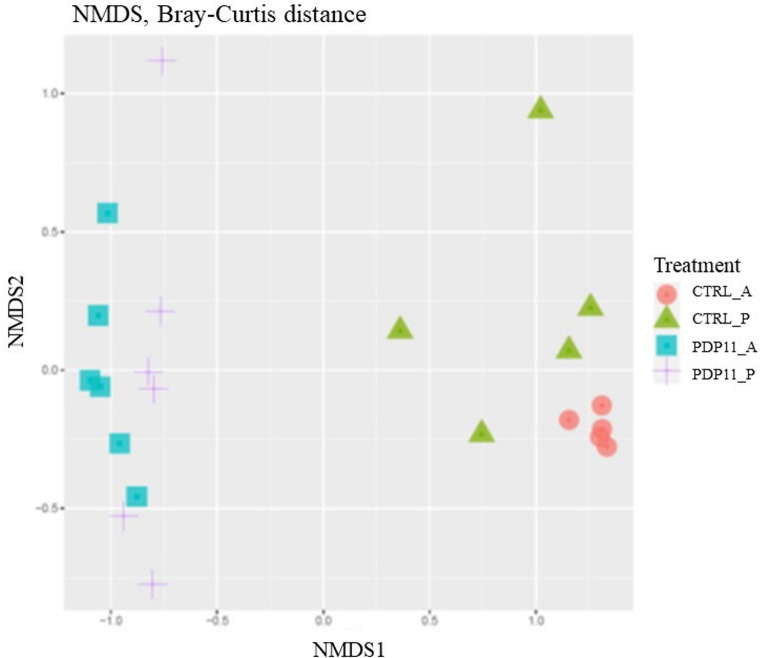
NMDS of bacterial community composition at ASV level based on Bray–Curtis distance matrix. Individual samples are color-coded based on the diets administered to. *S. senegalensis* specimens. Anterior (A) and posterior (P) intestinal microbiota of Senegalese sole specimens fed Control (CTRL) and sonicated probiotic SpPdp11 supplemented (PDP11) diets

A beta diversity analysis was conducted to investigate variations in bacterial communities according to the diet received by the fish. Visualization through NMDS plots revealed a clear separate clustering of intestinal samples associated to the diet (Fig. 4). Furthermore, the ANOSIM test corroborated the changes produced by adding a postbiotic to the diet in anterior and posterior intestinal microbiota (*p* < 0.05).

**Fig. 5:**
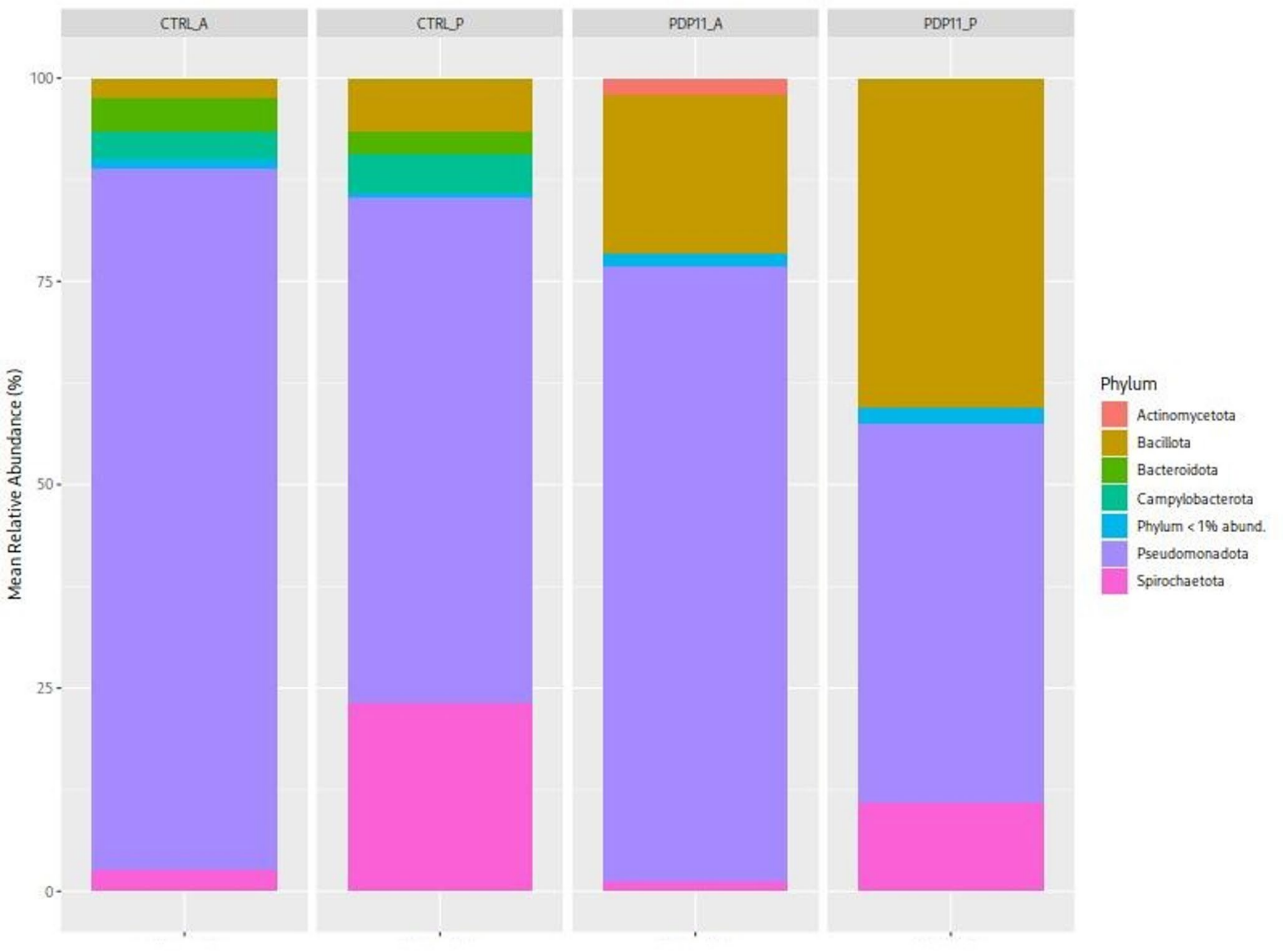
Bar plot of relative abundances at the phylum level by sample in the gastrointestinal tract of *Solea senegalensis*. Anterior (A) and posterior (P) intestinal microbiota of Senegalese sole specimens fed Control (CTRL) and sonicated probiotic SpPdp11-supplemented (PDP11) diets

*Pseudomonadota* was the most predominant phylum in anterior and posterior intestine of fish fed the control diet, while *Spirochaetota* was in the posterior section (Fig. 5). In fish fed the postbiotic diet, *Pseudomonadota* remained the most abundant phylum in both anterior and posterior intestinal sections, although its relative abundance was lower than in the CTRL group. This decrease was associated with an increase in Bacillota (Fig. 5).

At class level, *γ-Proteobacteria* was predominant in all experimental groups and sections (Supplementary Fig. 4). The abundance of *Bacilli* was higher in the posterior intestine of fish fed the postbiotic diet respect to the control; in contast, the abundances of *Campylobacteria* and *Brevinematia* were reduced, especially in the posterior section.

**Fig. 6:**
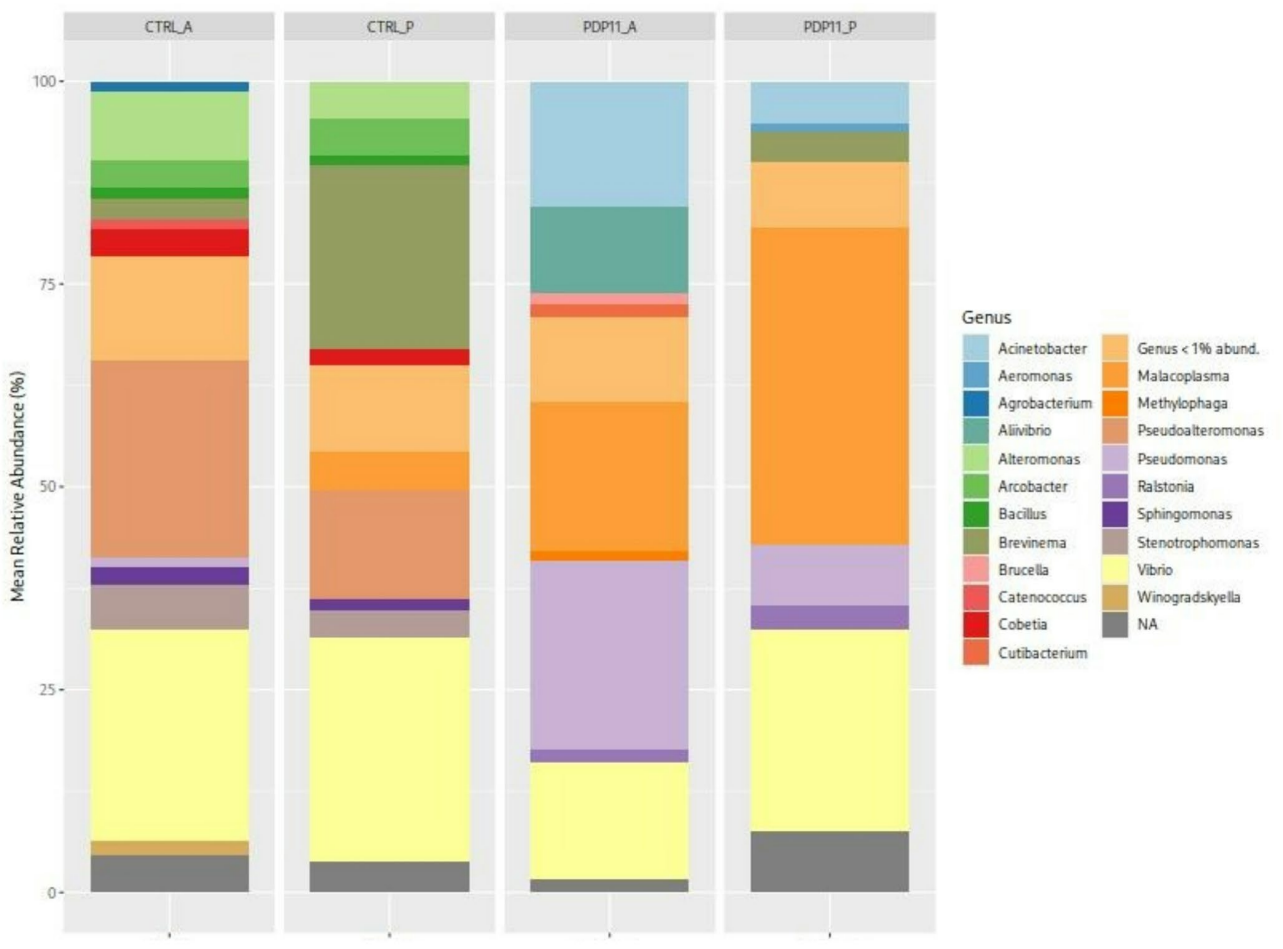
Bar plot of relative abundances at the phylum level by sample in the gastrointestinal tract of *Solea senegalensis*. Anterior (A) and posterior (P) intestinal microbiota of Senegalese sole specimens fed Control (CTRL) and sonicated probiotic SpPdp11-supplemented (PDP11) diets

**Fig. 7:**
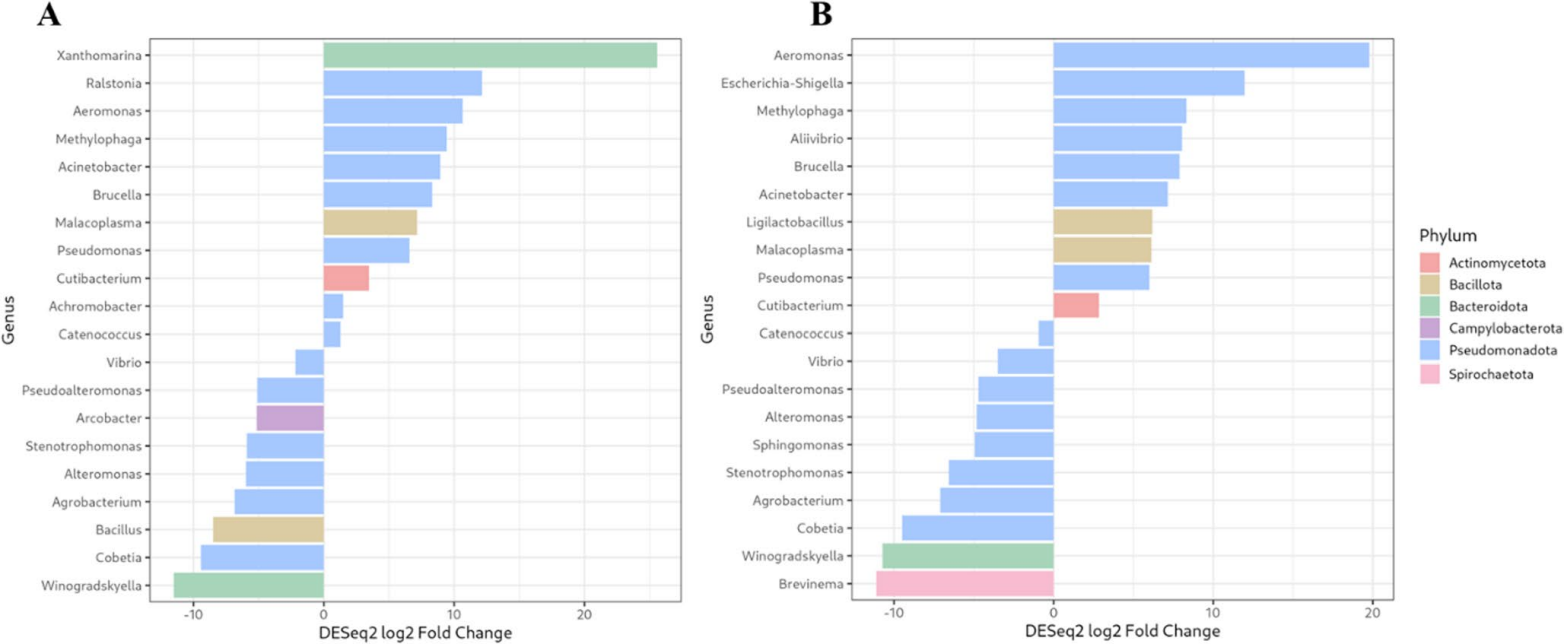
Significant differences (*p* < 0.05) in the 20 most abundant bacterial genera identified by DESeq2 between treatments in the intestinal microbiota of Senegalese sole fed the control diet (negative log₂ fold change) and the sonicated probiotic SpPdp11-supplemented diet (positive log₂ fold change). Each color represents the phylum to which each genus belongs. (**A**) Anterior intestine; (**B**) posterior intestine

The taxonomic analysis at genus level (Fig. 6) revealed a significant reduction in the relative abundance of genera such as *Alteromonas*, *Bacillus*, *Brevinema*, *Cobetia*, *Pseudoalteromonas*, *Stenotrophomonas*, and *Vibrio* in the intestinal microbiota of fish fed the postbiotic diet. Conversely, PDP11 group showed a significant increase in the abundance of *Acinetobacter*, *Malacoplasma* (formerly known as *Mycoplasma*), *Pseudomonas*, and *Ralstonia* (Fig. 7).

## Discussion

Maintaining intestinal health is critical in aquaculture for optimizing nutrient absorption, growth, and disease resistance (Dawood 2021). In this study, we demonstrate that dietary supplementation with sonicated SpPdp11 induces beneficial changes in the intestinal status of *S. senegalensis*, including improved tissue morphology, anti-inflammatory transcriptomic signatures, and modulation of the gut microbiota. These findings support the potential of this postbiotic strategy as a preventive tool to promote gut health and reduce disease susceptibility in aquaculture systems.

Morphometric measurements were conducted on anterior and posterior and they showed a significant higher *villus* width (VW) in the anterior intestinal section, a decreased epithelium height (EH), and a lower *lamina propria* thickness (LP), both intestinal sections in specimens fed on PDP11 dietary treatment. Nutrient absorption is carried out by intestinal *villi*, playing the enterocytes a crucial role in the digestion and absorption process (Hu et al. 2016). Dietary protease supplementation in the diet *Oreochromis niloticus* increased the height and width of intestinal *villi* (Saleh et al. 2022) suggesting a high absorption of nutrients and crucial factor for maintaining a healthy intestine (Abozeid et al. 2021). In the same sense, it was found that increased width and height of intestinal *villi* promoted increased growth performance in Nile tilapia when fish were fed a diet supplemented with gamma aminobutyric acid (Ruenkoed et al. 2023). In the present study, fish receiving the postbiotic diet also increased the diameter of the *villus* (VW) observed in the anterior intestinal section. As well as the higher values in *villi* height, might suggest an enhancement of the intestinal surface area for more efficient nutrient absorption compared to the control group. However, *villus* height (VH) marginally tended to increase in fish fed diets supplemented with sonicated SpPdp11 cells, though no significant different with control group was evidenced. Several authors reported that increased *villus* height by dietary changes positively correlated with better growth performance in fish (Araújo et al. 2016) (Bae et al. 2020). On the contrary, Nile tilapia fed diet formulated with 10% *Gracilaria* sp. showed significant reduction of *villi* length and lower growth (Silva et al. 2015). In the present study, no differences were found in *villi* height (VH) which supported the fact that similar growth were observed in both experimental groups.

Intestine is an important part that develops the innate immune system to prevent pathogen infections and inflammation, and healthy intestinal mucosa plays a crucial physiological role in promoting animal growth, digestion, and absorption functions. Taking this last into account, the *lamina propria* thickness (LP) is a parameter used as indicator of inflammatory process of connective tissue of the intestinal mucosa due to the infiltration of high number of leukocytes (Vizcaíno et al. 2014). Results obtained evidenced a noticeable reduction in the *lamina propria* thickness (LP) in both intestinal segments after dietary administration of sonicated SpPdp11. The reduction of thickness of intestinal submucosa layer by dietary administration of probiotic such as LAB has been also reported. Thus, the administration of *Lactobacillus brevis* and *L. buchneri* to *Seriola dumerili* receiving a diet with vegetable oils replacing fish oil reduced the thickness of *lamina propria* (Milián-Sorribes et al. 2021). In addition, Nimalan et al. 2023 also observed that the supplementation of a soybean meal diet with *L. plantarum* and *L. fermentum* reduced the *lamina propria* width, suggesting a relationship with the prevention of enteritis in specimens of *Salmo salar*. All these studies were assayed with live probiotic cells, but our study is the first focused on describing the effects of postbiotics applied to fish leading the same effect but using sonicated probiotic *Shewanella* sp. SpPdp11 cells.

Histological changes observed in the intestinal samples are related to the information obtained from the analysis of the *S. senegalensis* gut transcriptome receiving the postbiotic diet. Firstly, in comparison with anterior intestinal section, no significant functional enrichment of genes was observed in the posterior intestinal region of the fish fed with the diet including sonicated probiotic cells. This finding suggests that, although modifications in numerous genes occurred, they were not sufficiently intense to significantly alter the intestinal functionality. On the other hand, the lower pro-inflammatory status of the *lamina propria* may be related with the down-regulation of genes corresponding to the extracellular matrix (ECM) in the anterior intestinal section of fish receiving the postbiotic diet. The extracellular matrix is continuously remodelled by creating newly synthesised proteins such as collagen, laminins and elastin. ECM has profound influences on the structure, viability, and functions of cells, although certain fragments derived from the extracellular matrix exhibit the capacity to attract inflammatory cells. ECM-derived peptides have been reported to have chemotactic activity for inflammatory cells including collagen types I and IV, elastin, fibronectin, laminins, entactin/nidogen, thrombospondin, and hyaluronan (Adair-Kirk and Senior 2008; Castillo-Briceño et al. 2010). Then, excessive ECM remodelling has been linked to chronic intestinal inflammation in humans (Mortensen et al. 2019).

A significant reduction of the transcription of genes encoding PPAR, a nuclear receptor playing a crucial role in regulating lipid metabolism (Wahli and Michalik 2012). PPAR is abundantly present in the gastrointestinal tract, especially in epithelial cells (Mukherjee et al. 1997). Furthermore, the production of LPS by bacteria stimulates the expression of PPARγ and activates NF-κB (Wahli 2008). Considering that NF-κB is instrumental in regulating the response to stress or bacterial antigens, a decrease in PPAR levels could indicate a reduction in infectious processes or stress-related responses.

The primary bile acid pathway, overexpressed in diet supplemented with postbiotics, is intricately connected to cholesterol, which plays a crucial role in cell membrane composition and serves as a precursor to significant secondary metabolites such as sterols. It can be obtained from the diet or synthesised *de novo*, and the conversion of cholesterol to bile acids represents one of the main pathways for the elimination of excess cholesterol from the body (Kortner et al. 2014). Studies indicate that incorporating high levels of plant protein into fish diets can stimulate cholesterol biosynthesis, suggesting a potential inadequacy in cholesterol supply for fish (Geay et al. 2011; Kortner et al. 2014). In the results of this study, the upregulation of genes involved in the primary bile acid pathway in *S. senegalensis* was observed. It may indicates a potential improvement in the cholesterol assimilation pathway. Insufficient cholesterol levels were found to compromise immunity by suppressing both innate and adaptive immune component and, exacerbated the inflammatory response by increasing the expression of pro-inflammatory cytokines and concurrently decreasing the expression of anti-inflammatory cytokines (Wang et al. 2019). In addition, bile acids serve as physiological detergents, accelerating the absorption and transportation of lipids, vitamins, and nutrients by activating nuclear receptors, which, in turn, regulate metabolism and contribute significantly to overall health (Romano et al. 2020). Wang et al. (2023b) observed that inclusion of exogenous bile acids resulted in promoting growth, alleviating lipid accumulation, improvement of antioxidant capacity and immunity, and rebalanced intestinal microbiota.

The lipid metabolism is regulated by two key factors such as bile acids and gut microbiota (Claesson et al. 2012; Yoshimoto et al. 2013). In the case of mammals and fish there is increasing evidence for a close correlation between those two factors (Ridlon et al. 2006; Yokota et al. 2012; Liu et al. 2016). Bile acids exert a dual influence on the gut microbiota, as antimicrobial agents secreted into lumen of the intestine (Kurdi et al. 2006), and indirectly inducing antimicrobial peptides and lectins via bile acids receptors (Inagaki et al. 2006; D’Aldebert et al. 2009). Additionally, the gut microbiota play a role to biotransform the bile acids by degrading bile salts by deconjugation, bile acids oxidation, and epimerization (Ridlon et al. 2006, 2014). Thus, gut microbiota is instrumental in regulating the levels and composition of bile acids in various tissues (Swann et al. 2011), having a potential to change host physiology and metabolism. For these reasons, the microbiota analysis can be very relevant.

In this study, the microbial analysis showed a significant reduction in alpha diversity indices (Observed, Shannon index) in fish fed the postbiotic diet in comparison with the specimens of CTRL diet, demonstrating the capability of the sonicated cells of SpPdp11 to modulate the intestinal microbiota. In line with these results, Acosta et al. (2022) a reported reduction of the alpha diversity indices of intestinal tract of specimens of *S. senegalensis* was reported when the diet was supplemented with vitamin K1. It is thought that a lower Shannon index diversity can negatively affect the functionality of the intestinal microbiota (Noor et al. 2010; Falony et al. 2016; Lyons et al. 2017b), but it has also been reported that a high number of interacting species can often lead to destabilization (Finegold et al. 2010; Ponnusamy et al. 2011; Lozupone et al. 2013; Jiang et al. 2015). In agreement with this premise, in our study the values of Simpson’s index were not significantly different. Dominance is related to ecosystem functions, affecting process rates through species identity (the dominant trait) and evenness (the frequency distribution of traits), and indirectly influences the relationship between process rates and species richness (Hillebrand et al. 2008; Lozano and Rillig 2020).

*Pseudomonadota*, and γ-*Proteobacteria*, were the most predominant phylum and class respectively, in both intestinal sections. This result is in agreement with those obtained in previous studies in *S. senegalensis* (Tapia-Paniagua et al. 2019; Acosta et al. 2022) In one study carried out with specimens of Senegalese sole fed with a diet supplemented with heat-inactivated cells of SpPdp11 the predominance of *Pseudomonadota* was only observed in the anterior intestinal sections, whereas in the case of posterior intestine reported *Spirochaetota* as the most predominant phylum (Domínguez-Maqueda et al. 2021). *Pseudomonadota* includes Gram-negative bacteria with metabolic capabilities highly flexible and containing lipopolysaccharide (LPS) which induce the release of pro-inflammatory cytokines (Du et al. 2022). Tran et al. (2018) reported that an increase of *Pseudomonadota* in Grass carp is associated with unstable gut microbiota, and their increase is a potential diagnostic criterion for dysbiosis and disease. The significant reduction of the abundance of this phylum observed in fish fed the postbiotic diet, it could be suggested that it could be related with the better fitness status of the *lamina propia* observed in these specimens receiving the diet with sonicated cells of SpPdp11and with the down-regulation of the transcription of genes of routes such as ECM receptor interaction and PPAR Signalling Pathway which are involved in inflammatory processes.

At genus level, the diet supplemented with sonicated cells of SpPdp11 induced significant alterations in the microbial composition in the anterior and posterior intestinal sections of *S. senegalensis* specimens such as the significant increase of abundances of *Acinetobacter*,* Pseudomonas*, *Malacoplasma* (formerly known as *Mycoplasma*), and *Ralstonia*, genera commonly found in the digestive tracts of aquatic animals (Lyons et al. 2017a; Egerton et al. 2018). Although some species of *Pseudomonas* are considered pathogenic (Derome et al. 2016), other have been employed as probiotic organisms in aquaculture due to their ability to interfere with pathogenic microorganisms (Das et al. 2006; Liu et al. 2015). Regard it, strains of *P. fluorescens* has been used as a probiotic to combat bacterial pathogens such as *P. anguilliseptica* and *Streptococcu*s *faecium* in Nile tilapia (Eissa 2014), and one strain of *P. monteilii* from fish gut has demonstrated antimicrobial activity, with its major metabolite, 1-hydroxyphenazine, exhibiting efficacy against *Aeromonas hydrophila* (Qi et al. 2020).

It has been documented that *Malacoplasma* is more prevalent in healthy *Salmo salar* individuals than in diseased ones, with a positive correlation between its abundance and fish weight, suggesting a potential symbiotic relationship between the microorganism and its host (Bozzi et al. 2021). An important role of this genus have also been suggested for lipid metabolism in salmonids (Rasmussen et al. 2023). Then, it has revealed that within the gut microbiota of rainbow trout, sequences associated with unclassified *Mycoplasma* species were found to be more prevalent in *Flavobacterium psychrophilum*-resistant lines in comparison to susceptible fish (Mora-Sánchez et al. 2020). *Pseudomonas* species are known to form biofilms and usually they require a vitamin B12 as cofactor for their enzymatic activity (Fang et al. 2017). Notably, it has been found high levels of vitamin B in salmons is associated with an increased presence of *Mycoplasma* strains (Rasmussen et al. 2023). It is consistent with the results of this study, because fish fed the postbiotic-supplemented diet exhibited a higher abundance of both *Pseudomonas* and *Malacoplasma*, and it could suggest a potential interplay between this microorganism shifts and their vitamin metabolism.

*Acinetobacter*, in turn, showed a significant higher abundance in the in anterior section of specimens of PDP11 group. *Acinectobacter* has been recently reported as crucial role in the core microbiota of skin and gills of *Sparus aurata* (Cerezo et al. 2024). Conversely, members of *Actinobacteria* group are capable of producing butyrate (Parada Venegas et al. 2019). Butyrate, a short-chain fatty acid (SCFA), is known to have significant and proven beneficial effects, even in fish species as *S. aurata* (Estensoro et al. 2016). On the other hand, a significantly increased abundance of *Ralstonia* genus was also observed in fish fed postbiotic diet strains of this genus have shown antimicrobial activity and the capability of biosynthesis of beneficial secondary metabolites for the host (Jami et al. 2015; Cerezo-Ortega et al. 2021).

In addition, fish fed the postbiotic diet exhibited a significant reduction in the relative abundance of several genera commonly associated with opportunistic or potentially pathogenic bacteria such as *Pseudoalteromonas*, *Stenotrophomonas* and *Vibrio*) (Pujalte et al. 2007; Abraham et al. 2016; Maqbool et al. 2024)It has been reported that infections by pathogenic species of *Vibrio* caused by pathogenic species of this genus were associated with a decrease in the relative abundance of *Bacillota* and *Verrucomicrobiota* and increased *Pseudomonadota* (Kim et al. 2023). On the contrary, in this study, the dietary inclusion of sonicated cells of SpPdp11 significantly reduced the abundance of *Vibrio* a decrease in phyla *Pseudomonadota* and a concurrent increase in *Bacillota*. These decreases in *Vibrio* and *Pseudomonadota* abundances could suggest a potential effect of the postbiotic capability of sonicated cells of SpPdp11 on bacterial taxa related to intestinal poor health. However, the complex interactions among species and their potential substitution likely reflect an adaptive response to dietary changes aimed at maintaining ecosystem stability and functional consistency.

Taken together, the structural, molecular, and microbial findings indicate that dietary postbiotics derived from sonicated SpPdp11 cells can enhance intestinal health and reduce inflammation without the need for viable bacteria. This has important implications for aquaculture: postbiotics are inherently safer, more stable, and easier to incorporate into feed than live probiotics. From a preventive medicine perspective, this approach may help reduce the incidence of intestinal disorders, improve resilience against stressors, and potentially lower the need for therapeutic interventions in intensive farming.

## Conclusion

The results of this study demonstrate that dietary supplementation with sonicated SpPdp11 cells exerts measurable benefits on the intestinal health of *S. senegalensis*. Histological analyses revealed structural improvements indicative of reduced inflammatory status, including wider villi and thinner lamina propria. These findings were supported at the molecular level by the downregulation of pro-inflammatory signaling pathways and the upregulation of genes involved in bile acid metabolism. Furthermore, the postbiotic diet induced a clear modulation of the gut microbiota, reducing the abundance of pro-inflammatory or potentially pathogenic taxa (*Pseudomonadota*, *Vibrio*, *Stenotrophomonas*), and increasing beneficial genera such as *Acinetobacter*, *Pseudomonas*, *Malacoplasma*, and *Ralstonia*. These outcomes suggest that **postbiotics derived from sonicated SpPdp11 represent a safe and effective preventive strategy** to support gut health, modulate immune function, and promote microbial balance in aquaculture species. Unlike live probiotics, postbiotics offer improved stability, ease of feed integration, and reduced risk of unintended microbial interactions. This approach may serve as a **practical**,** scalable solution** to enhance fish welfare and resilience in intensive aquaculture systems, contributing to more sustainable and health-focused production practices.

## Supplementary Information

Below is the link to the electronic supplementary material.

Supplementary Material 1 (DOCX 56.0 KB)

Supplementary Material 2 (DOCX 50.7 KB)

Supplementary Material 3 (DOCX 53.3 KB)

Supplementary Material 4 (DOCX 46.4 KB)

Supplementary Material 5 (PNG 182 KB)

## Data Availability

The raw Fastq files are presently accessible through the National Centre for Biotechnology Information (NCBI), under the respective Bioprojects PRJNA1074949 (for *S. senegalensis* RNA data) and PRJNA1075492 (for 16 S bacterial ribosomal sequences).
