# Supplementary material for: Postbiotic Dietary Supplementation with Sonicated Shewanella sp. SpPdp11 Improves Intestinal Status in Juvenile Senegalese Sole (Solea senegalensis)
Source: Mar Biotechnol (NY). 2026 Apr 14;28(2):62. doi: 10.1007/s10126-026-10608-3 (PMC13079515; doi:10.1007/s10126-026-10608-3)
Supplement: Supplementary file 2 — Supplementary Material 2 (DOCX 50.7 KB) [file 10126_2026_10608_MOESM2_ESM.docx]

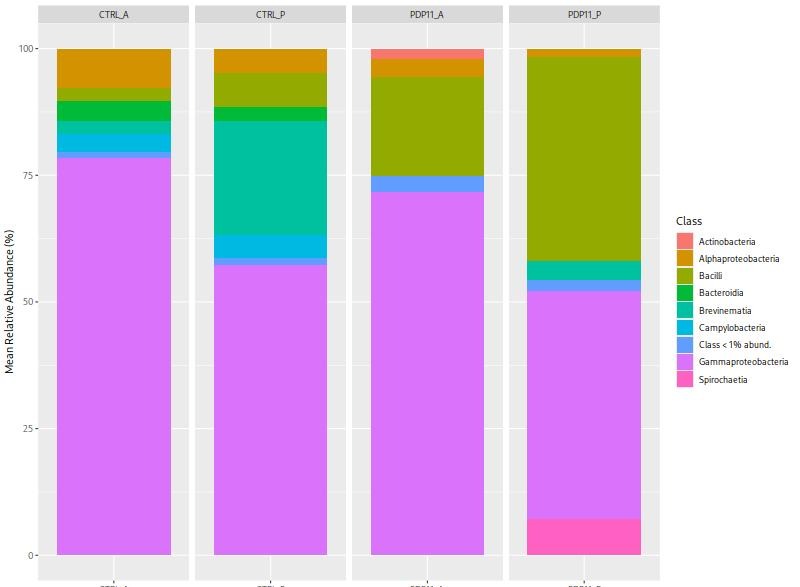


**Figure S4**. Barplot of class level relative abundances mean in the gastrointestinal tract of *S. senegalensis* fed control (CTRL) and *SpPdp11* supplemented (Pdp11) diet with sections labelled as A (anterior) and P (posterior). Values below 1% are indicated as Family < 1% abund.
