# Supplementary material for: Postbiotic Dietary Supplementation with Sonicated Shewanella sp. SpPdp11 Improves Intestinal Status in Juvenile Senegalese Sole (Solea senegalensis)
Source: Mar Biotechnol (NY). 2026 Apr 14;28(2):62. doi: 10.1007/s10126-026-10608-3 (PMC13079515; doi:10.1007/s10126-026-10608-3)
Supplement: Supplementary file 3 — Supplementary Material 3 (DOCX 53.3 KB) [file 10126_2026_10608_MOESM3_ESM.docx]

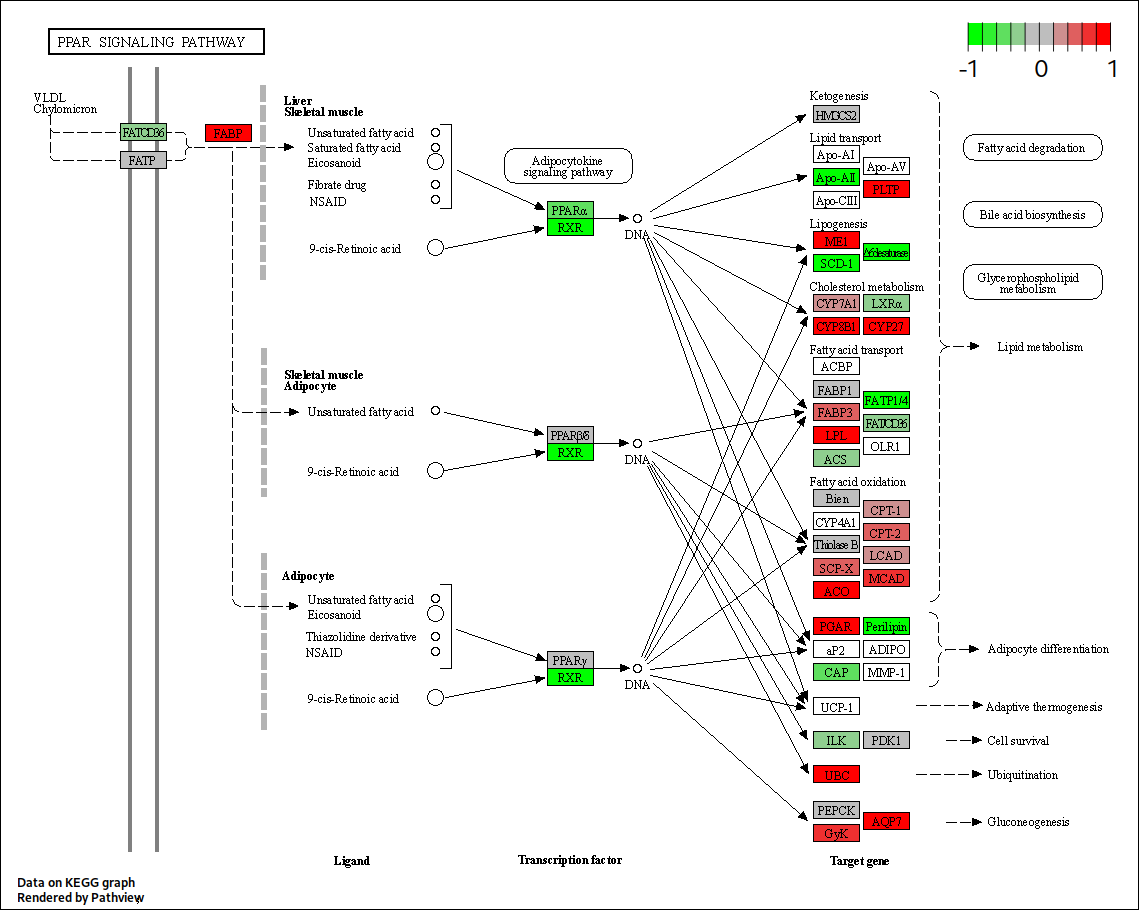


**Figure S2**. KEGG map of PPAR Signaling pathway analysis. Genes exhibiting significant overexpression are colored in red, while those showing repression are marked in green.
