# Supplementary figures and images for: Postbiotic Dietary Supplementation with Sonicated Shewanella sp. SpPdp11 Improves Intestinal Status in Juvenile Senegalese Sole (Solea senegalensis)
Source: Mar Biotechnol (NY). 2026 Apr 14;28(2):62. doi: 10.1007/s10126-026-10608-3 (PMC13079515; doi:10.1007/s10126-026-10608-3)

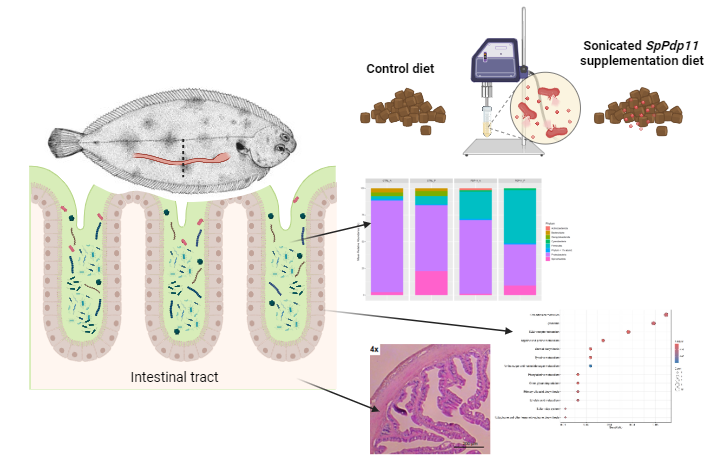

Supplement: Supplementary file 5 — Supplementary Material 5 (PNG 182 KB) [file 10126_2026_10608_MOESM5_ESM.png]
